# Dimensional Characterization and Hybrid Manufacturing of Copper Parts Obtained by Atomic Diffusion Additive Manufacturing, and CNC Machining

**DOI:** 10.3390/ma17061437

**Published:** 2024-03-21

**Authors:** Elena Monzón, Pablo Bordón, Rubén Paz, Mario Monzón

**Affiliations:** Mechanical Engineering Department, Universidad de Las Palmas de Gran Canaria, Edificio de Ingenierías, Campus de Tafira Baja, 35017 Las Palmas, Spain; elena.monzon@ulpgc.es (E.M.); mario.monzon@ulpgc.es (M.M.)

**Keywords:** additive manufacturing, hybrid manufacturing, atomic diffusion additive manufacturing, material extrusion additive manufacturing, dimensional characterization, CNC machining

## Abstract

The combination of Atomic Diffusion Additive Manufacturing (ADAM) and traditional CNC machining allows manufacturers to leverage the advantages of both technologies in the production of functional metal parts. This study presents the methodological development of hybrid manufacturing for solid copper parts, initially produced using ADAM technology and subsequently machined using a 5-axis CNC system. The ADAM technology was dimensionally characterized by adapting and manufacturing the seven types of test artifacts standardized by ISO/ASTM 52902:2019. The results showed that slender geometries suffered warpage and detachment during sintering despite complying with the design guidelines. ADAM technology undersizes cylinders and oversizes circular holes and linear lengths. In terms of roughness, the lowest results were obtained for horizontal flat surfaces, while 15° inclined surfaces exhibited the highest roughness due to the stair-stepping effect. The dimensional deviation results for each type of geometry were used to determine the specific and global oversize factors necessary to compensate for major dimensional defects. This also involved generating appropriate over-thicknesses for subsequent CNC machining. The experimental validation of this process, conducted on a validation part, demonstrated final deviations lower than 0.5% with respect to the desired final part, affirming the feasibility of achieving copper parts with a high degree of dimensional accuracy through the hybridization of ADAM and CNC machining technologies.

## 1. Introduction

Additive Manufacturing (AM) has undergone significant growth in recent decades, driven by a range of advantages over more conventional technologies. Among others, AM has facilitated manufacturing cost reduction, enabled mass customization, lowered manufacturing times primarily for small and intricate parts, and addressed highly complex geometries in a single piece [1,2,3]. The development of AM technologies for metallic materials has, moreover, succeeded in extending these advantages to sectors of extreme demand such as the aeronautical and aerospace industries, where turbine components, fuel injection systems, and even high-tech satellite components are already being produced [4,5]. In the biomedical sector, various types of metallic prostheses, bone regeneration scaffolds, or tissue engineering have been developed [5,6,7]. On the other hand, a significant number of components manufactured through AM have been implemented in the automotive industry, reducing costs and weight [8,9]. Furthermore, even the production of more efficient and complex energy batteries is now possible thanks to AM [10,11,12].

In the field of metal additive manufacturing technologies, classified according to ISO/ASTM 52900:2021 standards [13], a distinction can be made between those based on the use of metal powder—Directed Energy Deposition (DED), Powder Bed Fusion (PBF), or Binder Jetting (BJT)—and those primarily driven by metal–polymer filaments, such as Material Extrusion (MEX). The former stand out for their dimensional precision (PBF), the ability to generate highly complex geometries (PBF, BJT), or high productivity (DED, BJT) [14,15,16]. However, all of this entails high costs in terms of investment, operation, and the handling of complex and hazardous powders [15,17]. In contrast, MEX technologies are more cost-effective due to their filament extrusion system [18,19], but they also significantly limit the manufacturing resolution or complexity of the parts [14,15,20].

This is the case of the Atomic Diffusion Additive Manufacturing (ADAM) technology, developed by Markforged, which is based on the extrusion of various types of filaments composed of metal powder bound with waxes and polymeric resins. The production of fully metallic functional parts with this technology involves three consecutive manufacturing stages. First, an advanced material extrusion system deposits the filament layer by layer, similar to non-metallic MEX technologies, resulting in “green parts” composed of metal and its binders. In this phase, a second ceramic filament is also extruded by an additional extruder to prevent contact between the layers of the part material and the supports. Subsequently, in a chemical immersion washing system, specific solvents dissolve the soluble components of the binders. Afterwards, a drying process is applied, thus obtaining porous and fragile parts referred to as “brown parts”. In the final stage of the system, a thermal sintering device removes all the remaining binders and facilitates the atomic diffusion of metal particles, reducing final porosity and densifying the part to achieve densities close to those of a standard material [21,22]. However, this technology also has significant limitations that affect the surface and dimensional quality. On the one hand, an automatic oversizing of 15 to 20% must be carried out to compensate for the high contractions during the sintering phase [22,23], and the extrusion technology is currently limited to layer resolutions in the order of 0.1 mm [24,25]. It is necessary to incorporate support structures for overhanging surfaces due to the low mechanical stability of the material during the sintering process. Furthermore, the final properties can be highly anisotropic due to the effects of generation, densification, and closure of internal pores during the sintering process, as well as the orientation in which the parts are manufactured, the orientation of the filler particles, and the spaces found between extruded filaments [15,26].

To overcome these types of limitations, metal additive manufacturing technologies can be integrated and complemented with other technologies. This is the case with hybrid manufacturing (HM), where the customization capabilities and complexity of geometries of additive manufacturing are complemented by the high dimensional accuracy and surface quality of subtractive technologies such as computer numerical control (CNC) machining [27,28,29]. However, these hybridizations require an extensive analysis for optimizing the numerous variables associated with both technologies, which vary significantly from one another, in addition to those introduced by the material to be manufactured. Previous studies have examined optimal over-dimensioning and parameterization for laser-based powder bed fusion (PBF-LB) and 5-axis milling of Ti6Al4V material, resulting in significant improvements in surface quality after hybridization, except in holes or channels where direct machining remains the preferable option [30]. The variables, such as laser power, welding speed, or bead distance, have also been analyzed in the hybridization of machining and wire welding technology using CO_2_ laser radiation [31], or injection molds have been successfully produced through selective laser cladding (SLC) and subsequent milling [32]. Other successful examples of hybridization have been achieved with hybrid plasma deposition and milling (HPDM), obtaining dimensional accuracies in the order of 0.05% and a surface roughness of 2 μm [33].

On the other hand, various artifacts have been employed for the dimensional analysis of AM. Some authors have developed custom artifacts with multiple geometries to analyze the dimensional accuracy and corresponding standard tolerances according to ISO 286-1:1988 [34]. In other cases, dimensional analysis has been applied to specific parts with real applications [23,30] or to artifacts proposed by national organizations [35,36]. Dimensional tolerances in 17-4PH stainless steel artifacts without standardization have also been characterized using ADAM technology, obtaining IT12 and IT13 tolerances [22], as well as surface roughness values close to laser-sintered components [37]. In order to provide uniformity to this type of study, ISO/ASTM 52902:2019 [38] has defined a set of artifacts for assessing the dimensional and geometric accuracy of AM processes. These artifacts have already been employed to investigate additive manufacturing technologies with polymeric materials [39,40] or metallic materials in powder bed fusion [41], although no studies have been found involving standardized artifacts with ADAM technology.

Finally, and in the specific case of ADAM technology, there is still a broad field of study not only with materials like stainless steel, for which there are only a few studies on dimensional or mechanical characterization [22,34,42,43], but also with other industrially relevant materials such as copper. Additive manufacturing of copper parts is the subject of numerous studies [44,45] due to the combination of the advantages of additive manufacturing and the suitability of the material for both electrical [46,47] and thermal applications [48,49]. Despite this, there are virtually no studies on ADAM with copper material, and even fewer on dimensional analysis, with only a few references related to mechanical characterization and roughness [50] and electrical discharge machining [21]. Also, to the best of our knowledge, no hybrid manufacturing study has been conducted using ADAM technology, especially with copper as the material of interest. Therefore, this work presents a comprehensive and novel study of dimensional and surface characterization of copper parts obtained through Atomic Diffusion Additive Manufacturing (ADAM) technology, utilizing standardized test artifacts in accordance with ISO/ASTM 52902:2019. Furthermore, a dimensional analysis has been conducted for hybrid manufacturing to enhance the accuracy of parts through CNC machining processes.

## 2. Materials and Methods

### 2.1. Overview of the Hybrid Manufacturing Process

**Figure 1:**
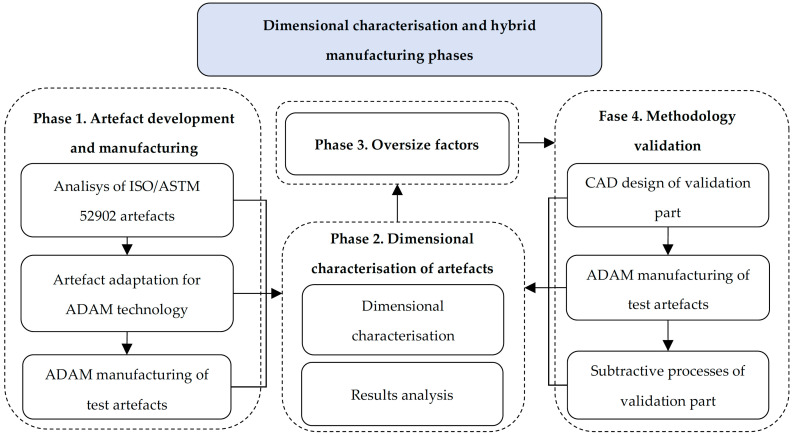
Diagram of the different phases of the methodology.

The development of the hybrid manufacturing process from parts obtained by ADAM technology was carried out through four main phases (Figure 1). Initially, and due to the limitations of ADAM, a comparative study was conducted between test artifacts from ISO/ASTM 52902:2019 and the manufacturing constraints outlined in the ADAM Metal X technology design guide [51], allowing for the adaptation of the artifacts and their subsequent fabrication. In a second stage, the artifacts were characterized by analyzing the dimensional deviations between the measurements taken on the pieces and their nominal design value. This characterization enabled the establishment, in the third phase of the study, of the different oversizing requirements necessary for both compensating deviations and subsequent machining in hybrid manufacturing.

**Figure 2:**
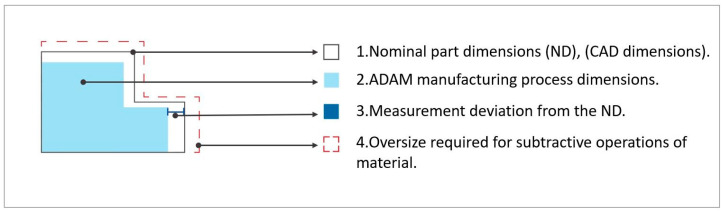
Schematic representation of dimensional changes in a component during the hybrid manufacturing process.

Finally, the methodology was validated through the hybrid manufacturing (ADAM and machining) of an oversized validation part, dimensionally characterizing its dimensional changes throughout the entire process (Figure 2).

### 2.2. Test Artifacts

For a comprehensive dimensional characterization of the parts obtained by ADAM technology, the 7 test artifacts defined in ISO/ASTM 52902:2019 have been employed. It is worth mentioning that after the experimental completion of this work, the updated standard (ISO/ASTM 52902:2023 [52]) was published, which includes some additional test artifacts. These artifacts enable the analysis of a broad set of dimensional aspects through standardized geometric elements. 

As precision components, the standard defines *Linear* and *Circular artifacts*. The *Linear artifact* consists of cubes with progressively increasing linear distances, while the *Circular artifact* comprises two concentric circular rings of different thicknesses. For resolution quality analysis, 4 additional artifacts are defined for both circular and straight geometries. The *Resolution Pin artifact* consists of 5 cylinders of varying height and diameter, whereas the *Resolution Hole* artifact is composed of a similar pattern but with circular holes. The resolution analysis of linear geometries is conducted using the *Resolution Rib artifact*, consisting of vertical ribs of constant height but varying thickness, and the *Resolution Slot artifact*, which consists of vertical partitions of equal height and thickness progressively spaced to create different slot widths. As a variation of the latter artifact, the standard defines a *Resolution Slot* with angularity, adding progressive height and angularity to the partitions. Finally, the analysis of surface texture is performed using a last artifact composed of 6 tabs manufactured with different angularity, ranging from 0 to 90° in 15° intervals (*Surface Texture artifact*). It is noteworthy that each of these artifacts (except for the *Linear artifact*) is arranged in three dimensional grades: Fine, Medium, and Coarse, resulting in a total of 19 artifacts. Due to the limitations of ADAM technology, a comparative analysis was conducted to assess the feasibility of manufacturing all these geometries based on the ADAM design guide. Table 1 summarizes the original standardized artifacts, their geometric purposes, the limitations encountered for ADAM fabrication, and the adaptations made to ensure their proper fabrication. In order to optimize the total number of artifacts to be studied, some of these adaptations include the consolidation of all geometries into a single artifact, even when they belong to different dimensional grades. Digital models of the modified artifacts are included as Supplementary Materials (Archive S1).

### 2.3. Validation Specimen

**Figure 3:**
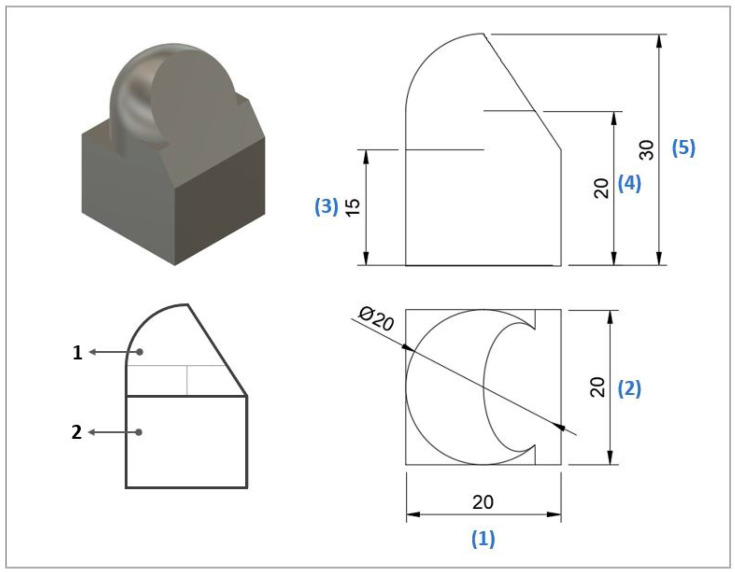
Validation specimen: General model, numeration, and dimensions in mm of primary geometries.

For the validation of the developed hybrid manufacturing process, a verification part was designed, as shown in Figure 3, consisting of spherical, cylindrical, horizontal, vertical, and inclined flat surfaces (56.3°). These shapes cover a wide range of geometries and facilitate the measurement process to verify the effectiveness of hybrid manufacturing. The required nominal dimensions after hybrid manufacturing are numbered from 1 to 5 according to Figure 3.

For the ADAM manufacturing of the validation specimens, the calculated oversize factors explained in Section 3.6 were applied, increasing the nominal dimensions. Since the surfaces corresponding to the specimen base served as anchoring areas and were not machined, they were not dimensionally characterized (dimensions 1 and 2). Nevertheless, dimension 2 was measured over the specimen in the cylindrical area machined, allowing for the characterization of these dimensions after the complete hybrid manufacturing process.

### 2.4. Materials and ADAM Equipment

The artifacts and the validation parts were manufactured with the ADAM technology Markforged Metal X^TM^ System (Markforged, Inc., Waltham, MA, USA). This material extrusion (MEX) system produces 3D printed parts (green parts) through the deposition of a filament material (metal powder in a plastic matrix). Subsequently, a chemical debinding process partially removes the wax and polymer binders (brown part). Finally, a sintering process in a furnace with inert atmosphere (argon and hydrogen) compacts the material to obtain a full dense metallic part. During the sintering process, approximately 16% shrinkage (for copper material) occurs. The manufacturing of artifacts and validation parts utilized Markforged copper filament F-MF-1010. Markforged ceramic release F-MF-1002 Type-1 filament served as the support material during the printing stage, and Opteon SF79 Specialty Fluid solvent (The Chemours Company, DE, USA) was employed for the washing step. The final sintered pieces were composed of 99.8% Cu, 0.05% oxygen, and 0.05% iron, in addition to other negligible components [53].

Additive manufacturing was configured with a post-sintered layer height of 0.129 mm, a solid fill pattern, 4 wall layers (1.03 mm post-sintered), and a melt temperature of 220 °C. In general, the test artifacts were manufactured with a raft-type platform, except for the cases of *Resolution Rib* and *Resolution Pin* artifacts due to their design featuring a broad base. Additionally, for a concise comparative analysis of the potential effect of using a raft and interior fill in the artifacts, a second *Circular artifact* was fabricated without a raft, as well as a third artifact without a raft and with a non-solid triangular fill (triangular grids, which reduce the time and material required for manufacture). As the last artifact, a second variant of *Resolution Hole* was manufactured in a vertical orientation (with respect to the printing base) to analyze the holes generated vertically, following the recommendations of ISO/ASTM 52902:2019. Regarding the validation pieces, they were manufactured solid and without a raft as the design featured a solid base. 

**Figure 4:**
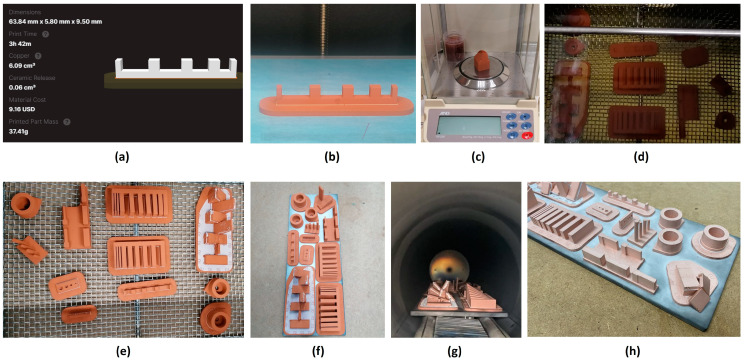
Manufacturing processes using ADAM technology: (**a**) 3D printing configuration; (**b**) 3D printing (green part); (**c**) Weighing; (**d**) Debinding; (**e**) Drying; (**f**) Brown parts; (**g**) Sintering; and (**h**) Final parts.

Eleven test artifacts (Figure 4) and three validation pieces were manufactured, requiring a total of 169 h of printing time and approximately 2 kg of copper filament. After the washing and drying process, all pieces met the minimum mass reduction requirement of 2.7%, in accordance with the ADAM manufacturing procedure.

### 2.5. Subtractive Manufacturing

**Figure 5:**
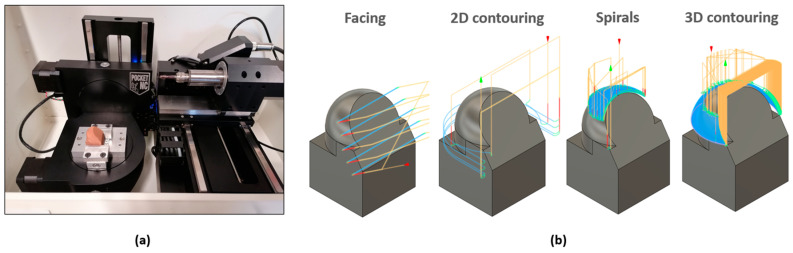
Machining process: (**a**) 5-Axis CNC Machining Equipment: Pocket NC V2-50; and (**b**) Machining operations.

The subtractive process involved machining through milling, employing a 5-axis CNC machining system (Figure 5) with the 5-axis Pocket NC V2-50 (Penta Machine Co., Belgrade, MT, USA), suitable for high-speed machining of soft and small-sized materials. It has a linear and angular resolution of 6.1 μm and 0.01° respectively, a lineal repeatability of ±50.8 μm, an angular repeatability of ±0.05°, a spindle runout of 2.5 μm, and a manufacturing tolerance of ±127 μm. Machining operations were carried out with a 4 mm diameter solid carbide spherical end mill (model 2452K) and a 6 mm diameter solid carbide flat end mill (model 2444K), both from EMUGE-Werk Richard Glimpel GmbH & Co. KG (Nürnberger, Germany). The tools were secured using a Nakanishi CHK-6 collet (Nakanishi Inc., Utsunomiya, Japan). CNC milling control programming was carried out using the CAM module within Fusion 360 software v2.0 (Autodesk, Inc., San Francisco, CA, USA).

Several preliminary tests were conducted to select the best cutting conditions, the clamping system, finishing of validation parts, and the numerical control code programming for machining. These tests involved the gradual adjustment of both cutting speed and feed rate to establish proper machining conditions for the tools used and the material being machined (copper). In addition, diverse workpiece clamping positions were scrutinized, mirroring conventional machining practices. Furthermore, basic tests were conducted to validate the simulated numerical control sequences and to determine appropriate stepovers for the desired geometries and operations, among other parameters. The primary parameters ultimately set for machining the validation piece are summarized in Table 2.

### 2.6. Dimensional and Roughness Characterization

The dimensional characterization process required the use of diverse metrological equipment. Measurement of the most accessible linear dimensions was carried out using a digital height gage (Mitutoyo Corporation, Kawasaki, Japan) and a digital caliper (JBM CAMPLLONG, Girona, Spain), both with a resolution of 0.01 mm. For less accessible dimensions, a Nikon V-12A Profile Projector with an SC-112 digital counter (Nikon Corporation, Tokyo, Japan) with a 1 μm resolution, and an Olympus BX51 optical microscope (Olympus Corporation, Tokyo, Japan), were employed. Ten measurements were taken for each dimension under characterization.

For roughness characterization, the Surface Texture artifact from Table 1 was utilized, with each tab extracted from the base of the artifact. Five roughness readings were taken on the upper face of each tab and along its length, except for the tab manufactured at 90°, where the measured surface corresponds to its larger dimension lateral face. Arithmetical mean roughness (Ra) and maximum height (Ry) measurements were taken using a Mitutoyo SJ-201P surface roughness tester (Mitutoyo Corporation, Kawasaki, Japan), accompanied by data acquisition software, following ISO 1997 guidelines [54]. The measurements included a 2.5 mm cut-off length, 3 sampling lengths, and profile R with a 2CR75 filter.

## 3. Results

### 3.1. Manufacturing Defects of the Test Artifacts

**Figure 6:**
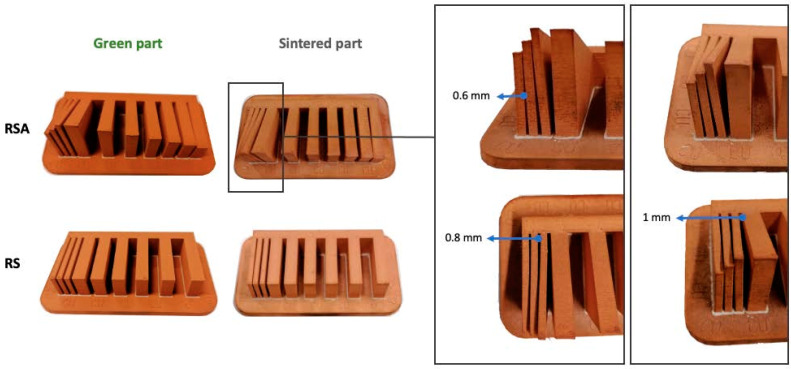
Test artifacts of Resolution Slot, both in the pre-sintered (green part) and post-sintered (sintered part) states. Deformation of the slots with angularity in 2 mm thick walls after the sintering process.

After the complete fabrication of the artifacts, and before proceeding with their dimensional characterization, a set of dimensional and manufacturing defects were observed in specific geometries. The *Resolution Slot* with angularity (RSA M-C) artifact exhibited deformation at the end of the 2 mm thick partitions (the minimum value recommended by the design guide of the technology). The same artifact without angularity (RS M-C) also exhibited this deformation in these 2 mm geometries, albeit to a lesser extent, showcasing the influence of the height and angularity deformation on the corners of such geometries (Figure 6).

In reference to the RP-C *Resolution Pin* artifacts, the rods with a height-to-diameter ratio of 6:1 (RP-C-LD6, with diameters of 4 mm and 3 mm) exhibited inclination defects after the final sintering process, thereby indicating the dimensional constraints associated with such slenderness. 

**Figure 7:**
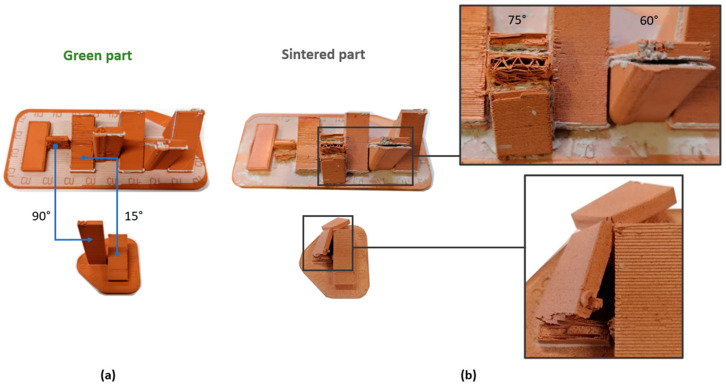
Additive manufacturing defects in Surface Texture artifacts: (**a**) Green part; (**b**) Sintered part; breakage of the 90° and 75° part after sintering, and separation of the supports from the 60° part.

Regarding manufacturing defects, the *Surface Texture (ST)* artifact generated several flawed geometries (Figure 7). During the printing phase, tabs with 90° and 15° angles exhibited irregularities between deposited layers, resulting in almost complete detachment of the deposited filament in the 90° tab and causing overlapping defects in the 15° tab. This necessitated the printing of a new independent artifact with 90° and 15° tabs. Following the sintering process, both the 90° and 75° tabs detached, leading to the collapse of both, while the 60° inclined tab exhibited pronounced deformation and separation from its support structure. Both effects may be induced by the slender nature of the geometries and the differential contraction between solid areas (final part) and supports (non-solid triangular infills). These defects highlight the challenge and limitation of the technology in generating slender geometries, emphasizing the influence of support structures and the ceramic interface separating the part from the support.

### 3.2. Results of the Dimensional Characterization of the Test Artifacts

Due to the substantial number of geometries analyzed in each test artifact, the results are presented in an aggregated manner for each of them, despite having geometries with different nominal dimensions. This involves averaging the outcomes across sets of similar geometries, even when they possess varying nominal dimensions. To do so, the mean values and their standard deviations for all the dimensions analyzed for each artifact under study were calculated. In a first step, for each artifact and type of geometry (lengths, heights, thicknesses, cylinder diameters, hole diameters, and slot widths), the mean dimensional deviation (in absolute terms) was obtained separately, by Equation (1), comparing the mean value of each geometry measured with the nominal dimension.

(1) $$DDG\;\left(mm\right)=\frac{\sum \left(GM(mm)-ND\left(mm\right)\right)}{n1}$$

With *DDG* being the dimensional deviation of the geometry, *GM* the geometry measurement, *ND* the nominal dimension, and *n*1 the number of measurements.

In a second step, and using Equation (2), all the deviations calculated for each geometry with Equation (1) were used to obtain an average artifact deviation (in absolute terms as well). Additionally, the percentage deviation was calculated using Equation (3).

(2) $$DDA\;\left(\mathrm{mm}\right)=\frac{\sum \left(DDG\;(\mathrm{mm})-ND\;\left(\mathrm{mm}\right)\right)}{n2}$$

(3) $$PD\left(\%\right)=\frac{DDA\left(mm\right)}{ND(mm)}\cdot 100$$

With *PD* being the percentage deviation of the artifact, *n*2 the number of geometries, and *DDA* the dimensional deviation of the artifact.

For example, in the case of the *Resolution Hole* artifact (comprising four holes of different nominal dimensions), the average deviations for each hole were calculated, followed by determining the overall average deviation (absolute and percentage) for the entire set of holes.

All these results are presented in Table 3. In the initial columns, the deviations of the artifacts after the MEX process (green parts) are depicted. These deviations range between 8.91% and 31.19%, indicating a lack of linearity between similar geometries. This is evident in the percentage deviations, where the deviations are normalized relative to the nominal value. This fact confirms the complexity of the ADAM technology and the influence of geometry type on the manufacturing process. It is also noteworthy that deviations in the vertical extrusion axis are significantly higher (18.27% in *Resolution Rib* height and 18.66% in *Resolution Pin* height) than deviations of geometries parallel to the printing plane (13.44% in *Resolution Rib* thickness and 8.91% in *Resolution Pin* diameter), likely due to effects during sintering caused by the weight of softened material. Additionally, it should be noted that these large percentage deviations in the green parts were to be expected as the slicer software (Eiger v3.17) automatically oversizes the green part according to the material used (with no control possibilities in this respect) to counteract the shrinkage that will occur during the sintering process (binder removal and densification of the part). For this reason, all the percentage deviations of the green parts are positive (larger 3D printed parts than the nominal dimensions), with an overall value of about 16%.

Regarding the results of the sintered parts (after the entire manufacturing process), most geometries exhibited deviations below 0.148 mm, except for the *Linear artifact* (0.224 mm) and the *Resolution Pin* artifact (0.464 mm in height). Moreover, a predominant trend in the deviations leans toward the positive side, resulting in geometries larger than the nominal design of the part, except for the non-solid *Circular artifact* without raft (−0.027 mm) and the *Resolution Rib* (thickness measurement), where the average deviation was negative (−0.03 mm), indicating a value lower than the nominal.

The analysis of the dimensional deviations in a percentage format (normalized according to the nominal design dimension of each geometry) reveals a greater disparity in the results, with no uniformity or deviation pattern observed. It is noteworthy that deviations from small-diameter circular geometries (*Resolution Hole* and *Resolution Pin* diameters) exceed 4% deviation (especially in the resolution hole artifact with vertical orientation, with 5.04% deviation due to the stair-stepping effect [55]), while most linear geometries show values below 2%. This could be attributed to the higher potential for error in measuring small-diameter circular geometries, as well as the increased difficulty in executing these geometries using ADAM technology. On the other hand, it should be noted that the standard deviations of the percentage deviations in the medium-grade resolution slot specimens were particularly high due to the deformations of the specimens in the sintering process.

Regarding the variation in the use of raft and the type of infill in the CA-F Circular artifact, raft elimination and the use of non-solid infill apparently showed the best dimensional accuracy, with the supported artifact exhibiting the highest deviations. However, the differences were low and further studies would be necessary to statistically verify the influence of these manufacturing process variables.

The existence of positive deviations (geometries with dimensions greater than the nominal ones) and negative deviations (geometries with dimensions smaller than the nominal ones) can lead to compensation when calculating average values, resulting in aggregated average deviations lower than the actual ones. Therefore, Table 4 shows the corrected average values based on the absolute values of the deviations. Only those geometries whose results are affected by this compensation are shown (geometries not displayed maintain the results from Table 3).

Taking into account this correction, a slight increase is observed in the *Circular artifact* and *Linear artifact* deviations, and a drastic increment in the defective warped geometries of the *Resolution Slot* with angularity (13.3% and 38.3% deviation). Therefore, these latter geometries will be excluded from the subsequent stages of analysis.

The standard deviation calculated for the mean values generally indicates a high degree of dispersion in the data. It is important to note that these average values have been derived from various geometries with differing nominal dimensions. At this stage, a tentative average value has been calculated to provide a general perspective.

### 3.3. Results of Surface Texture Characterization

**Figure 8:**
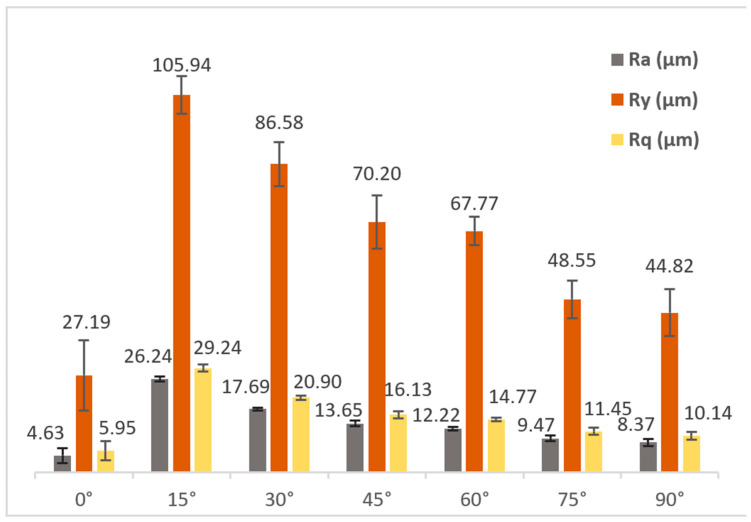
Results of roughness measurements (Ra, Ry and Rq) for each manufacturing angle of the test artifact Surface Texture ST-M.

In Figure 8, the surface roughness results of the *Surface Texture* artifact are summarized. The lowest roughness values were achieved on the horizontal surfaces (as expected, as the stair-stepping effect will be noticeable in the rest of angles, especially for low angles), with Ra roughness values of 4.63 ± 2.03 µm (although with some result dispersion), aligning with the findings from studies with stainless steel and ADAM technology [18,22]. The surface inclination at 15° significantly worsened the surface roughness, reaching the highest values in the entire series (26.24 ± 0.69 µm due to the stair-stepping effect). The progressively increasing inclination led to a continuous improvement in roughness, reaching the second-best result at 90° inclination (8.37 ± 0.96 µm). It is worth noting that inclinations of 45° and 60°, commonly used in MEX additive manufacturing to avoid the use of support structures, produced similar roughness values. From a hybrid manufacturing perspective, these results highlight the need for generating allowances for subsequent finishing machining of surfaces, especially in geometries with lower inclination angles. For this purpose, it is recommended to oversize surfaces with values exceeding the maximum profile depth (Ry) shown in Figure 8. 

### 3.4. Analysis of the Manufacturing Orientation of Circular Holes

**Figure 9:**
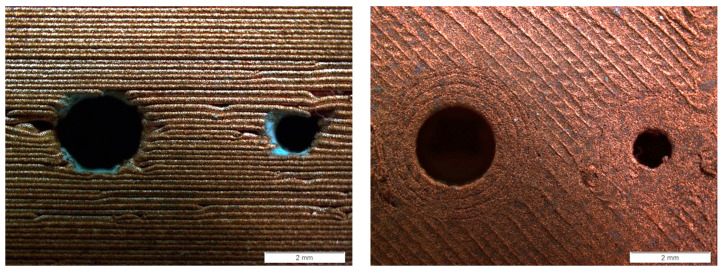
Optical microscopy image of 1 and 2 mm diameter holes in each artifact. Resolution hole: printing in vertical configuration (**left**) and printing in horizontal configuration (**right**).

In relation to the two Resolution Hole artifacts manufactured in horizontal and vertical orientations, the deviation results (Table 3) did not show very significant differences when constructing the holes in the vertical configuration (percentage deviation of 5.04% for vertical configurations against 4.07% for horizontal orientation), contrasting with the visual appearance of the artifacts. However, optical microscopy images reveal substantial irregularities in the vertically manufactured artifact, decreasing the roundness of the holes compared to the horizontally fabricated artifact (Figure 9). These irregularities primarily stem from inaccuracies in layer deposition during the material extrusion phase, both in the regions forming the piece and in the supports. The latter significantly affect the upper layers of hole closure, inadequately reproducing the final layers, particularly in the smaller-diameter holes. Furthermore, the incorporation of ceramic filament (essential for the separation between the piece and supports, both made of copper) introduces additional distortions due to inaccuracies in the automatic change of extrusion heads or the disparate behavior of materials during the sintering phase.

### 3.5. Analysis of Oversize Factors

The results presented in the previous sections provide a basic understanding of the minimum over-thickness required for subsequent machining in parts manufactured using ADAM technology. These over-thicknesses can be customized for each geometry in parts with well-defined zones and medium complexity. However, variations in deviations exist between types of artifacts with similar geometries, and resizing different geometries in complex parts can be challenging, potentially affecting the various oversize factors.

To establish a more general and practical approach to oversize, four specific oversize factors have been calculated based on the predominant geometry of the part to be obtained through hybrid manufacturing. For this purpose, the results of all the analogous measurements of the artifacts were grouped into four main dimensional categories: cylindrical in the horizontal manufacturing plane (XY), circular holes in the horizontal manufacturing plane (XY), linear in the horizontal manufacturing plane (XY), and linear in the vertical manufacturing direction (Z-axis). In this way, average dimensional and percentage deviations were calculated per dimensional category, rather than per artifact.

For the calculation of the cylindrical dimensional category, measurements of external cylindrical features in the *Circular artifact* and *Resolution Pin* were used. To characterize holes, both interior cylindrical geometries and holes in the *Circular artifact* and *Resolution Hole* were utilized. Concerning linear dimensions in the horizontal plane, measurements of linear features in various artifacts were used, along with new linear measurements taken on their bases or available linear geometries (except *Surface Texture*). Similarly, measurements available in the vertical axis (heights) of all artifacts (except *Surface Texture*) were used for the vertical linear category.

#### 3.5.1. Specific Oversize Factor for Cylindrical Geometries

Table 5 presents the set of measurements used to analyze cylindrical geometries, indicating the nominal dimensions of the artifact and the average deviations for each geometry. The average value of all deviations is shown in the last row, with a value of −0.069 ± 0.022 mm (−2.24 ± 0.26%), indicating that the generated geometries are smaller than the nominal dimension. However, only small-diameter cylinders exhibit negative deviations, as larger diameters predominantly deviate positively. For this reason, it is reasonable to differentiate the oversizing factors for both geometric groups, as shown in the last rows. Thus, the mean deviation for larger-diameter cylindrical geometries (23.5 and 25 mm) would amount to 0.018 ± 0.029 mm (0.08 ± 0.12%), and for smaller-diameter ones, it would be −0.155 ± 0.014 mm (−4.57 ± 0.41%). However, for a more conservative oversizing, the most unfavorable values found can be considered. Based on these data, oversizing of at least 0.5% is recommended for the larger geometries, solely to reach the nominal dimension of the original part in the most unfavorable situation encountered, along with an additional amount for subsequent machining. Similarly, for smaller-diameter cylindrical geometries, oversizing of at least 6% is recommended to compensate for dimensional deviations.

Regarding data dispersion, it can be observed that the deviations of the mean values are lower than those observed in Table 3, primarily due to the more specific geometries involved and the detailed analysis of the different geometries.

#### 3.5.2. Specific Oversize Factor for Circular Holes

The holes exhibited similar dimensional deviations across most diameters, implying a higher percentage deviation for smaller-diameter holes. Nonetheless, the overall trend indicates an increase in size relative to the nominal dimension (Table 6). Therefore, this reduction (negative oversizing) needs to be compensated for by incorporating additional thickness intended for subsequent machining.

For these geometries, an average deviation value of 0.129 ± 0.053 mm was obtained, with maximum deviations reaching less than 0.2 mm. The average percentage deviation amounted to 3.08%, with a standard deviation of 1.63%. Given that dimensional deviations are constrained within a narrow range, it is more representative to establish this value as a measure of negative oversize (undersize) than to use a single percentage value, as this would result in unequal dimensional correction among nominal dimensions. Therefore, a negative oversizing of −0.2 mm is recommended, solely intended for compensating the increase in nominal dimension, with an additional negative oversize for machining operations.

With regard to data dispersion, the deviations of the mean values exhibit patterns akin to those observed in cylindrical geometries, showcasing reasonable dispersion values, particularly within the *Resolution Hole* artifact.

#### 3.5.3. Specific Oversize Factor for Linear Dimensions in the Horizontal Printing Plane (XY Plane)

The deviations of linear dimensions in the horizontal printing plane (XY plane) exhibit some variability (Table 7), although with maximum values around 1 mm and negative deviations slightly below −100 µm, and with standard deviations slightly higher than those obtained in other geometries. Therefore, the obtained deviations are predominantly positive, with an average value of 0.228 ± 0.052 mm, which allows for the exclusion of any dimensional adjustments for compensating deviations in these geometries. Only the desired oversizing for the subsequent machining phase is established.

#### 3.5.4. Specific Oversize Factor for Linear Dimensions in the Vertical Manufacturing Direction (Z)

In the vertical manufacturing direction, all the obtained measurements exceeded the design dimension with average percentage deviations ranging from 0.95% to 2.84% (excluding the *Resolution Hole* artifact due to its anomalous deviation). This implies average dimensional deviations between 95 and 682 µm (Table 8). In terms of data dispersion, there is a general increase observed, likely attributed to heightened difficulty in measuring heights across the artifacts. Therefore, considering the inherent oversizing generated by the technology in this manufacturing direction (0.261 ± 0.159%), there is no need to incorporate additional allowances except for the desired machining oversize.

### 3.6. Overall Oversize Factor

The specific oversize factors established in the preceding sections allow for the compensation of dimensional deviations arising during manufacturing using ADAM technology. However, for a subsequent machining process, two additional factors must be considered: deviations caused by piece fixation defects and machining oversize.

To compensate for deviations and inaccuracies in the clamping process (machining clamping), an additional general oversizing of at least 1% of the nominal dimension is recommended, except in the case of circular holes where a diameter reduction is suggested with a non-percentage value of −0.2 mm. Regarding machining oversize, it is further recommended that an additional general value of 0.25 mm be established at each end for most geometries, except for circular hole types, where the oversize should again be negative, with −0.25 mm being sufficient for the characterized geometries (diameters between 1 and 15 mm). This oversizing value aligns with the machining depth ranges of similar studies, albeit on polymeric pieces where layer height influences the final finish, unlike ADAM technology due its sintering process [56]. These machining oversize values should be applied to each end of the nominal dimension when peripheral machining of the piece is required.

Table 9 summarizes all the oversize factors according to the type of analyzed geometry and the purpose of oversizing, along with the composite global factor resulting from the accumulation of individualized factors. Additionally, it presents the minimum and maximum oversizes obtained by applying this global factor to the smallest and largest nominal dimensions, respectively, for each geometric category.

### 3.7. Validation Specimen Results

According to the oversize factors established in Table 9, a 1.5% oversize would be applied to the cylindrical geometry of the validation model, with an additional 0.25 mm over-thickness at each end (0.5 mm in total) for subsequent machining. Regarding the rest of the linear geometries, a 1% oversizing would be applied, along with the machining oversize of 0.25 mm. However, the oversize for peripheral machining was increased to 0.5 mm at each end to ensure the final dimensions of the validator due to the previously unknown effectiveness of the methodology in this study and for the homogenization of the validation part dimensions.

**Figure 10:**
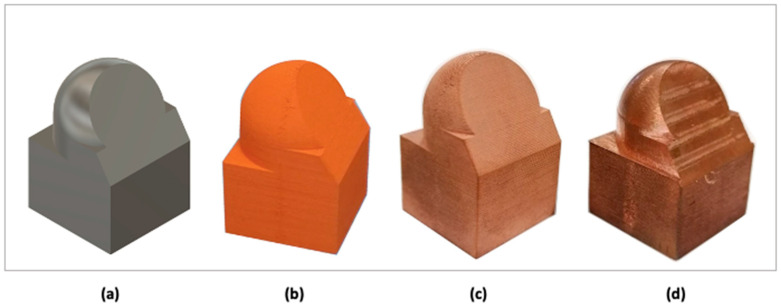
Stages of the hybrid manufacturing process for the validator: (**a**) Designed part; (**b**) 3D printed part (green part); (**c**) sintered part; (**d**) machined part.

The final oversizing incorporated into the design of the validation pieces (Figure 10a) is shown in the third column of Table 10, along with the results of dimensional characterization both after the sintering stage of the additive manufacturing process (Figure 10c) and after completing the hybrid manufacturing through CNC machining (Figure 10d).

The dimensional characterization of the validation parts exhibited satisfactory dimensional accuracy (repeatability) after the sintering process, given the low dispersion of mean values, with typical deviations ranging from 0.04 to 0.15 mm (Table 10). Regarding dimensional deviations, the mean deviations obtained with respect to the nominal dimension are considered compatible with the requirements of hybrid manufacturing, being very close to the expected dimensions (errors between −0.01 and 0.68 mm). Following the CNC machining process, programmed to achieve the nominal design dimensions of the validator, a high dimensional accuracy was achieved, with dimensional deviations between 0.04 and 0.09 mm, implying percentage deviations between 0.2 and 0.5% of the target dimension value.

**Figure 11:**
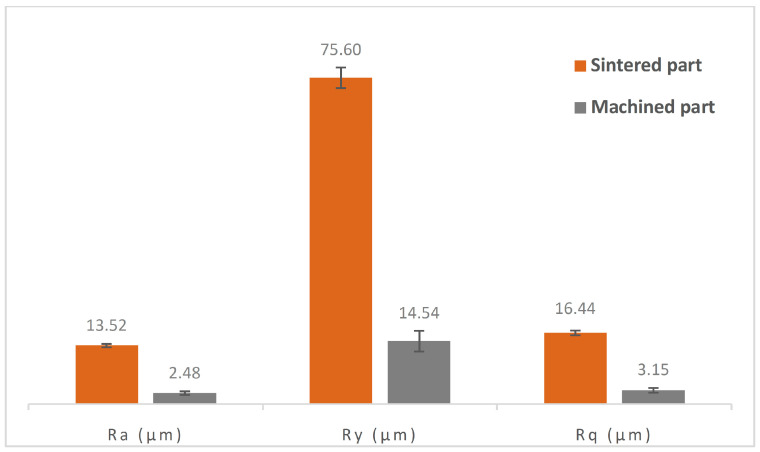
Mean roughness (Ra), root mean square roughness (Rq), and maximum profile depth (Ry), with the corresponding standard deviations, of the validation parts after the sintering and CNC machining processes.

The analysis of the surface texture of the validator (Figure 11) showed a significant reduction of the arithmetic mean roughness Ra, root mean square roughness Rq, and maximum profile depth Ry by more than 80% after the machining phase.

## 4. Conclusions

This work introduces a hybrid manufacturing methodology for copper metal parts, combining Atomic Diffusion Additive Manufacturing (ADAM) technology, dimensionally characterized through standardized test artifacts according to ISO/ASTM 52902:2019, with computer numerical control (CNC) machining, following the determination of a set of oversize factors. Furthermore, the methodology is validated through the application of the hybrid manufacturing method on a test piece.

The use of test artifacts (adapted to ADAM technology) allowed for a methodical characterization of a wide range of geometries and the identification of certain limitations, primarily observed in slender geometries, both cylindrical and flat with thicknesses of 2 mm. These geometries exhibited significant warping and even detachment during the sintering phase due to a lack of mechanical stability, even though these geometries fulfilled the design guidelines of the system.

Dimensional analysis revealed that in cylindrical geometries, nominal values should be increased with oversize factors to avoid obtaining effective dimensions lower than the design specifications. On the other hand, reductions in dimensions are required for circular holes since ADAM technology tends to oversize their diameters. Linear geometries such as lengths, thicknesses, or heights generate positive oversize, useful for subsequent machining, making it possible to omit dimensional compensation in this type of geometry within the framework of hybrid manufacturing.

Regarding surface texture, horizontal flat geometries exhibited the best average roughness, while inclinations of 15° resulted in the highest roughness (5.67 times higher), decreasing progressively as the inclination angle increased (stair-stepping effect). These roughness values can be improved by incorporating an oversize greater than the maximum profile depth values obtained at each inclination angle.

The application of the hybrid manufacturing methodology on a demonstrator piece, establishing oversize values calculated based on the main geometries, achieved dimensional accuracies of 0.5%, implying dimensional deviations in the hundredths of a millimeter for pieces obtained through MEX technology. This validates the potential of hybrid manufacturing using ADAM technology and the developed methodology.

Finally, it is important to note that the scope of this study is limited to the dimensional study of test artifacts manufactured in copper with dimensions up to 70 mm in length. Therefore, additional research would be required to validate the methodology for larger components and other materials used in ADAM technology. 

## Figures and Tables

**Table 1:** ADAM feasibility analysis of the test artifacts from ISO/ASTM 52902:2019. Proposed modifications.

Artifact Nomenclature and Dimensional Purpose	Original Artifact	ADAM Manufacturing Feasibility			Modified Artifact	Justification for Modification	Measured Dimensions
		Fine Grade (F)	Medium Grade (M)	Coarse Grade (C)			
***Lineal artifact (LA)*** Precision of linear positioning along a machine axis is assessed.	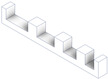	Non-existent ^1^	Yes	Non-existent ^1^	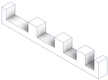	No modifications were applied.	-Position of the cube faces with respect to a part edge. Length of the part contour ^3^.
***Circular artifact (CA)*** Dynamic precision is studied to project the activation energy or the method of joining material onto the manufacturing surface in the AM machine.	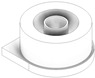	Partially	Partially	No ^2^	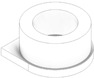	Removal of the inner ring due to a thickness below the minimum compatible with ADAM technology.	-Inner and outer ring diameters. Inner hole, base.
***Resolution pin (RP)*** The ability of an AM system or material to produce fine features with different aspect ratios is analyzed.	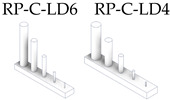	No	No	Partially	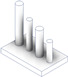	Removal of pins with diameters or lengths below ADAM technology limits and consolidation of M and C grades into a single artifact.	-Diameters of the pins. -Pin heights. -Length of the part contour ^3^.
***Resolution hole (RH)*** The minimum size of the internal cylindrical feature achievable at different aspect ratios is analyzed.	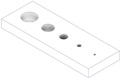	No	No	Partially	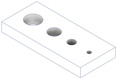	Removal of holes with diameters below the limits of ADAM technology.	-Diameters of the holes. -Length of the part contour ^3^.
***Resolution rib (RR)*** The minimum thickness achievable by an AM system is examined.	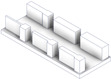	No	No	Partially	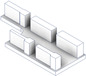	Rib removal with dimensions below ADAM technology limits.	-Thickness of the rib. -Rib height. -Length of the part contour ^3^.
***Resolution slot (RS)*** The minimum dimension of a slot or the minimum spacing between components is assessed.	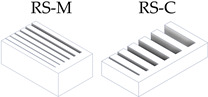	No	Partially	Partially	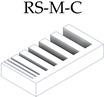	Removal of grooves with widths below the limits of ADAM technology and consolidation of M and C grades into a single artifact.	-Slot width. -Length of the part contour ^3^.
***Resolution slot with angularity (RSA)*** The minimum dimension of a slot or the minimum spacing between components is assessed.	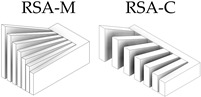	No	Partially	Partially	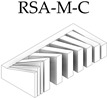	Removal of grooves with widths below the limits of ADAM technology and consolidation of M and C grades into a single artifact.	-Slot width. -Length of the part contour ^3^.
***Surface texture (ST)*** Surface texture of elements manufactured by AM systems is studied.	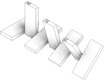	No	Yes	Yes	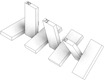	Given the adequacy of the medium grade, the coarse grade was not taken into consideration. No modifications were applied.	-Mean surface roughness.

^1^ Grade not specified by the standard. ^2^ The dimensions of the green part exceed those of the sintering furnace (Sinter-1). ^3^ As complementary measures for calculating the specific oversize factor for linear dimensions (Section 3.5.3).

**Table 2:** Machining process cutting conditions.

Operation	Area	Geometric Data	Spindle Speed (rpm)	Feed Rate (mm/min)	Stepover (mm)	Direction	Depth (mm) Roughing/Finishing
Facing	Inclined surface	15 mm height	2000	30	4.2	Climb milling	0.5/0.1
2D contouring	Cylindrical surface	5 mm height	5000	30	-	Climb milling	2.0/0.1
Spirals	Spherical surface	Up to 45° angle	4000	100	0.1	Climb-conventional	-/0.6
3D contouring	Spherical surface	From 45° angle	4000	100	0.1	Climb milling	-/0.1

**Table 3:** Results of the dimensional characterization of test artifacts without correction of values. Mean values and standard deviation (SD).

	Greeen Part				Sintered Part			
	Dimensional Deviation (mm)		Percentage Deviation (%)		Dimensional Deviation (mm)		Percentage Deviation (%)	
	Mean	SD	Mean	SD	Mean	SD	Mean	SD
**Circular artifact**								
Solid without raft	2.683	1.336	16.14	1.17	0.056	0.122	0.81	1.20
Solid with raft	2.691	1.384	15.96	0.62	0.130	0.063	1.37	1.56
Non-solid without raft	2.681	1.391	15.87	0.55	−0.027	0.051	0.05	0.60
**Resolution hole**								
Horizontal orientation	0.464	0.230	18.55	1.39	0.090	0.035	4.07	1.54
Vertical orientation	n/a	n/a	n/a	n/a	0.109	0.060	5.04	3.20
**Resolution slot**								
*Without angularity*								
Coarse grade	0.642	0.295	19.44	2.80	0.140	0.051	4.64	1.78
Medium grade ^1^	0.205	0.040	25.92	0.064	0.042	0.126	3.61	17.58
*With angularity*								
Coarse grade	0.683	0.334	20.15	0.064	0.143	0.131	2.62	4.84
Medium grade ^1^	0.246	0.045	31.19	2.59	0.059	0.454	0.33	50.06
**Resolution rib**								
Thickness	0.550	0.248	13.44	1.09	−0.030	0.016	−1.00	0.85
Height	1.827	0.020	18.27	0.20	0.148	0.065	1.48	0.65
**Lineal artifact**								
Face-to-face distance	4.350	3.039	15.01	2.05	0.224	0.248	0.15	1.71
**Resolution pin**								
Diameter	0.317	0.095	8.91	1.28	−0.155	0.015	−4.56	1.04
Height	3.269	0.966	18.66	0.35	0.464	0.154	2.64	0.21

^1^ Geometries with deformations after the sintering process. n/a: not applicable. Impossibility of measurement due to the presence of non-removable interior support until sintering.

**Table 4:** Results of the dimensional characterization of test artifacts with correction for negative values.

	Sintered Part			
	Dimensional Deviation (mm)		Percentage Deviation (%)	
	Mean	SD	Mean	SD
**Circular artifact**				
Solid without raft	0.098	0.079	0.97	1.02
Non-solid without raft	0.050	0.011	0.42	0.36
**Resolution slot**				
*Without angularity*				
Medium grade	0.100	0.058	13.3	7.80
*With angularity*				
Coarse grade	0.167	0.090	5.03	1.14
Medium grade ^1^	0.328	0.223	38.3	17.24
**Lineal artifact**				
Face-to-face distance	0.266	0.195	1.22	1.12

^1^ Geometries with deformations after the sintering process.

**Table 5:** Analysis of the oversizing factor for cylindrical geometries (sintered parts).

Artifacts and Geometries	Nominal Dimension (mm)	Mean Measurement (mm)		Dimensional Deviation (mm)		Percent Deviation (%)	
		Mean	SD	Mean	SD	Mean	SD
**Circular artifact without raft**							
Cylinder outer diameter	23.5	23.502	0.036	0.002	0.036	0.01	0.15
Base outer diameter	25.0	24.916	0.026	−0.084	0.026	−0.34	0.10
**Circular artifact with raft**							
Cylinder outer diameter	23.5	23.559	0.017	0.059	0.017	0.25	0.07
Base outer diameter	25.0	25.096	0.036	0.096	0.036	0.38	0.14
**Resolution pin**							
Ratio 6:1 y Ø 4 mm	4.0	3.867	0.019	−0.133	0.019	−3.33	0.47
Ratio 6:1 y Ø 3 mm	3.0	2.835	0.009	−0.165	0.009	−5.51	0.30
Ratio 4:1 y Ø 4 mm	4.0	3.837	0.012	−0.163	0.012	−4.08	0.29
Ratio 4:1 y Ø 3 mm	3.0	2.840	0.017	−0.160	0.017	−5.34	0.58
**Mean deviation of larger cylinders**				**0.018**	**0.029**	**0.08**	**0.12**
**Maximum deviation of larger cylinders**				**0.096**	**0.036**	**0.38**	**0.14**
**Mean deviation of smaller cylinders**				**−0.155**	**0.014**	**−4.57**	**0.41**
**Maximum deviation of smaller cylinders**				**−0.165**	**0.019**	**−5.51**	**0.30**
**Mean global deviation**				**−0.069**	**0.022**	**−2.24**	**0.26**

**Table 6:** Analysis of the oversize factor for holes manufactured in the XY plane (sintered parts).

Artifacts and Geometries	Nominal Dimension (mm)	Mean Measurement (mm)		Dimensional Deviation (mm)		Percent Deviation (%)	
		Mean	SD	Mean	SD	Mean	SD
**Circular artifact without raft**							
Cylinder outer diameter	15.0	15.192	0.048	0.192	0.048	1.28	0.32
Base hole diameter	5.0	5.114	0.011	0.114	0.011	2.28	0.22
**Circular artifact with raft**							
Cylinder outer diameter	15.0	15.184	0.028	0.184	0.028	1.23	0.18
Base hole diameter	5.0	5.180	0.012	0.180	0.012	3.61	0.23
**Resolution hole**							
Diameter 4 mm	4.0	4.081	0.012	0.081	0.012	2.04	0.29
Diameter 3 mm	3.0	3.120	0.004	0.120	0.004	4.01	0.13
Diameter 2 mm	2.0	2.115	0.008	0.115	0.008	5.74	0.40
Diameter 1 mm	1.0	1.045	0.020	0.045	0.020	4.48	2.00
**Maximum deviation**				**0.192**	**0.048**	**5.74**	**0.40**
**Mean global deviation**				**0.129**	**0.018**	**3.08**	**0.47**

**Table 7:** Analysis of the oversize factor for linear geometries manufactured in the XY plane (sintered parts).

Artifacts and Geometries	Nominal Dimension (mm)	Mean Measurement (mm)		Dimensional Deviation (mm)		Percent Deviation (%)	
		Mean	SD	Mean	SD	Mean	SD
**Resolution hole**							
Length horizontal orientation (axis X)	22.5	22.489	0.011	−0.011	0.011	−0.05	0.05
Length horizontal orientation (axis Y)	10	9.913	0.025	−0.087	0.025	−0.87	0.25
**Resolution slot without angle**							
Length (axis X)	58.4	58.869	0.101	0.469	0.101	0.80	0.17
Length (axis Y)	30	30.205	0.157	0.205	0.157	0.68	0.52
**Resolution rib**							
Length (axis X)	72	73.073	0.142	1.073	0.142	1.49	0.20
Length (axis Y)	28.5	28.794	0.047	0.294	0.047	1.03	0.16
**Lineal artifact**							
Length (axis X)	55	55.489	0.013	0.489	0.013	0.89	0.02
Length (axis Y)	5	4.874	0.015	−0.126	0.015	−2.52	0.30
**Resolution pin**							
Length (axis X)	28	28.258	0.022	0.258	0.022	0.92	0.08
Length (axis Y)	18	18.156	0.033	0.156	0.033	0.87	0.18
**Circular artifact without raft**							
Base outer diameter (axis X,Y)	25	24.916	0.026	−0.084	0.026	−0.34	0.10
**Circular artifact with raft**							
Base outer diameter (axis X,Y)	25	25.096	0.036	0.096	0.036	0.38	0.14
**Maximum deviation**				**1.073**	**0.157**	**−2.52**	**0.30**
**Mean global deviation**				**0.228**	**0.052**	**0.27**	**0.18**

**Table 8:** Analysis of the oversize factor for linear geometries manufactured in the vertical direction Z (sintered parts).

Artifacts and Geometries	Nominal Dimension (mm)	Mean Measurement (mm)		Dimensional Deviation (mm)		Percentage Deviation (%)	
		Mean	SD	Mean	SD	Mean	SD
**Lineal artifact**							
Artifact height	8.0	8.151	0.043	0.151	0.043	1.89	0.53
**Circular artifact without raft**							
Artifact height	13.0	13.289	0.035	0.289	0.035	2.22	0.27
**Circular artifact with raft**							
Artifact height	13.0	13.181	0.052	0.181	0.052	1.39	0.40
**Resolution hole**							
Height of artifact with horizontal orientation	2.5	2.862	0.048	0.362	0.048	14.48 *	1.90
Height of artifact with vertical orientation	10.0	10.194	0.022	0.194	0.022	1.94	0.22
**Resolution slot without angle**							
Artifact height	10.0	10.171	0.047	0.171	0.047	1.71	0.47
**Resolution rib**							
Height (6 mm thickness)	10.0	10.095	0.023	0.095	0.023	0.95	0.23
Height (5 mm thickness)	10.0	10.097	0.041	0.097	0.041	0.97	0.41
Height (4 mm thickness)	10.0	10.110	0.032	0.110	0.032	1.10	0.32
Height (3 mm thickness)	10.0	10.219	0.035	0.219	0.035	2.19	0.35
Height (2 mm thickness)	10.0	10.218	0.037	0.218	0.037	2.18	0.37
Height plus base	13.0	13.342	0.047	0.342	0.047	2.63	0.36
**Resolution pin**							
Pin height (6:1 y Ø 4 mm)	24.0	24.682	0.072	0.682	0.072	2.84	0.30
Pin height (6:1 y Ø 3 mm)	18.0	18.454	0.037	0.454	0.037	2.52	0.21
Pin height (4:1 y Ø 4 mm)	16.0	16.384	0.041	0.384	0.041	2.40	0.25
Pin height (4:1 y Ø 3 mm)	12.0	12.334	0.042	0.334	0.042	2.78	0.35
**Maximum deviation**				**0.682**	**0.072**	**2.84**	**0.30**
**Mean global deviation**				**0.261**	**0.041**	**1.98**	**0.34**

* Exclusion of an unusually high value from the analysis.

**Table 9:** Summary of specific and overall oversize factors for hybrid manufacturing based on ADAM-produced parts.

Geometry Type	Oversize Factors			Global Factor ^2^	Minimum Oversize (mm)	Maximum Oversize (mm)
	Specific (%)	Clamping (%)	Machining ^1^ (mm)			
Major cylinders	0.5	1.0	0.25	1.5% + 0.5 mm	0.85	0.88
Minor cylinders	6.0	1.0	0.25	7% + 0.5 mm	0.71	0.78
Circular holes	−0.2 mm	−0.2 mm	−0.15	−0.70 mm	−0.70	−0.70
Linear horizontal plane	0.0	1.0	0.25	1% + 0.5 mm	0.73	1.22
Linear vertical direction	0.0	1.0	0.25	1% + 0.5 mm	0.53	0.74
**Global (average)** **^3^**					**0.71**	**0.91**

^1^ At each end of the geometry if necessary. ^2^ Considering peripheral machining of the nominal dimension. ^3^ Excluding circular holes.

**Table 10:** Initial oversizing and results in validation specimens.

Dimension ^1^	Nominal Dimension (mm)	Oversize (mm)	Expected Dimension ^2^ (mm)	Sintered Part				Hybrid Manufacturing		
				Measurement (mm)		Deviation from Expected Dimension (mm)		Measurement (mm)		Dimensional Deviation ^3^ (mm)
				Mean	SD	Mean	SD	Mean	SD	
**1**	20.0	1.20	21.20	21.35	0.04	0.15	0.02	n/a	n/a	n/a
**2**	20.0	1.20	21.20	21.38	0.07	0.18	0.02	20.04	0.02	0.04
**3**	15.0	1.15	16.15	15.87	0.04	−0.28	0.01	15.08	0.06	0.08
**4**	20.0	1.20	21.20	21.19	0.15	−0.01	0.09	20.04	0.06	0.04
**5**	30.0	1.30	31.30	31.98	0.05	0.68	0.02	30.09	0.01	0.09

^1^ According to Figure 3. ^2^ After oversizing of the nominal dimension. ^3^ Related to the nominal dimension. n/a: Unmachined surface due to being a clamping zone during machining.

## Data Availability

The raw data supporting the conclusions of this article will be made available by the authors on request.

## Supplementary Materials

The following supporting information can be downloaded at: https://www.mdpi.com/article/10.3390/ma17061437/s1. Archive S1: Test artifacts modified.
